# Magnetic-Core–Shell–Satellite Fe_3_O_4_-Au@Ag@(Au@Ag) Nanocomposites for Determination of Trace Bisphenol A Based on Surface-Enhanced Resonance Raman Scattering (SERRS)

**DOI:** 10.3390/nano12193322

**Published:** 2022-09-24

**Authors:** Jie Huang, Tianxiang Zhou, Wenshi Zhao, Min Zhang, Zhibo Zhang, Wangsheng Lai, Naveen Reddy Kadasala, Huilian Liu, Yang Liu

**Affiliations:** 1 Key Laboratory of Functional Materials Physics and Chemistry of the Ministry of Education, Jilin Normal University, Changchun 130103, China; 2 Changchun Institute of Optics, Fine Mechanics and Physics, Chinese Academy of Sciences, Changchun 130033, China; 3 Department of Chemistry, Towson University, Towson, MD 21252, USA

**Keywords:** magnetic, core–shell–satellite nanocomposites, surface-enhanced resonance Raman scattering, coupling reaction, FDTD, bisphenol A

## Abstract

As a typical representative of endocrine-disrupting chemicals (EDCs), bisphenol A (BPA) is a common persistent organic pollutant in the environment that can induce various diseases even at low concentrations. Herein, the magnetic Fe_3_O_4_-Au@Ag@(Au@Ag) nanocomposites (CSSN NCs) have been prepared by self-assembly method and applied for ultra-sensitive surface-enhanced resonance Raman scattering (SERRS) detection of BPA. A simple and rapid coupling reaction of Pauly’s reagents and BPA not only solved the problem of poor affinity between BPA and noble metals, but also provided the SERRS activity of BPA azo products. The distribution of hot spots and the influence of incremental introduction of noble metals on the performance of SERRS were analyzed by a finite-difference time-domain (FDTD) algorithm. The abundance of hot spots generated by core–shell–satellite structure and outstanding SERRS performance of Au@Ag nanocrystals were responsible for excellent SERRS sensitivity of CSSN NCs in the results. The limit of detection (LOD) of CSSN NCs for BPA azo products was as low as 10^−10^ M. In addition, the saturation magnetization (Ms) value of CSSN NCs was 53.6 emu·g^−1^, which could be rapidly enriched and collected under the condition of external magnetic field. These magnetic core–shell–satellite NCs provide inspiration idea for the tailored design of ultra-sensitive SERRS substrates, and thus exhibit limitless application prospects in terms of pollutant detection, environmental monitoring, and food safety.

## 1. Introduction

Endocrine-disrupting chemicals (EDCs), also known as environmental hormones, are exogenous substances that affect mammalian reproduction by interfering with the endocrine system of organisms [1,2,3]. Bisphenol A (BPA), as a typical representative of EDCs, has been used principally in manufacturing polycarbonates and epoxy resins which are the main raw materials of plasticizers, plastic bottles and cups, food storage and packaging materials, and other commonly used industrial products [4,5]. Unfortunately, a large amount of evidence shows that the persistence, bioaccumulation, and biomagnification of BPA can bring about serious negative effects on human health and the ecosystem [6,7]. Although BPA is a low-toxicity chemical, it can induce a variety of diseases even at very low concentrations, such as congenital disabilities, diabetes, cardiovascular diseases, multiple cancers, and especially reproductive system diseases [8,9,10]. Hence, it is urgent to explore a rapid, sensitive, efficient, and low-cost detection method for BPA.

At present, direct detection and indirect detection are two commonly used methods for BPA detection. The direct detection method of BPA mainly includes high-performance liquid chromatography (HPLC), gas chromatography/mass spectrometry (GC-MS) and liquid chromatography/mass spectrometry (LC-MS) [11,12,13]. Nevertheless, these methods ordinarily need complex sample pretreatment, long-term testing, professional testers, and expensive equipment, which limit their practical application. By comparison, indirect detection methods, such as plasmon resonance biosensors and enzyme-linked immunosorbent assay, have high sensitivity [14,15]. However, the complex antibody preparation and the blurry recognition boundaries between specificity and non-specificity still need to be solved [16]. In recent years, as a highly sensitive spectral analysis technology, surface-enhanced Raman scattering (SERS) has stimulated considerable research enthusiasm due to its high sensitivity, easy operation, multiplexing ability, and applicability, and it is extensively used in the detection of chemical and biological molecules [17,18,19,20]. Both electromagnetic enhancement (EM) and chemical enhancement (CE) are widely recognized SERS enhancement mechanisms [21,22]. A large number of experimental results have proven that the EM mechanism shows an enhancement of 10^4^ to 10^11^, while the CE mechanism only contributes to 10 to 10^3^ enhancement [23]. Therefore, it is widely acknowledged that EM plays a key role in SERS enhancement [24]. Given that the enhancement of EM caused by localized surface plasmon resonance (LSPR) excitation usually occurs in nanogaps between metal nanoparticles (named “hot spots”), it is highly desirable to increase the number of “hot spots” for obtaining high performance SERS substrates [25,26,27].

Inducing aggregation and constructing interlayers are two commonly used methods for introducing high- density hot spots. Nonetheless, the introduction of aggregation will inevitably bring about non-uniform distribution of noble metals, which is not conducive to the purpose of increasing hot spots [28]. In comparison, the use of polymers to achieve the adsorption or self-assembly of hot spots on surface of composite SERS substrates has attracted more and more attention [29,30]. Polyethyleneimine dithiocarbamate (PEI-DTC) polymer with excellent bonding strength is an ideal interlayer, which can significantly improve the affinity of Au and Ag nanocrystals to the SERS surfaces due to the presence of the bidentate ligands with two chelating sulfur groups [31]. Unfortunately, single-element Au nanocrystals or Ag nanocrystals have limited plasmonic absorption [32]. The bimetallic Au@Ag nanocrystals can not only have richer plasmonic modes and tunable LSPR but also make full use of outstanding SERS activity of Ag nanocrystals as well as uniformity and stability of Au nanocrystals [33,34,35]. However, the affinity between phenolic molecules such as BPA and noble metals is rather weak when mixing the target analytes with noble metal substrates directly, which is bad for the acquisition of high-quality SERS signals [36]. Because azo dyes have a propensity to bind to noble metal nanocrystals, the coupling reaction of BPA and diazonium ions to produce azo dyes is an ideal solution for the SERS detection of BPA, which can greatly boost the activity of SERS substrates [37]. In addition, the resonance enhancement occurs when the excitation wavelength overlaps or approaches the electronic transition of target molecules, which is called surface-enhanced resonance Raman scattering (SERRS) [38]. Therefore, the combination of azo dyes derived from BPA and SERRS will make it possible for ultra-sensitive detection of BPA.

Herein, we propose magnetic-core–shell–satellite Fe_3_O_4_-Au@Ag@(Au@Ag) nanocomposites (CSSN NCs), which could serve as ultra-sensitive SERRS substrates for BPA detection. A simple and rapid coupling reaction of Pauly’s reagents and BPA was used not only to solve the problem of poor affinity between BPA and noble metals, but also to advance SERRS enhancement performance. BPA azo products were selected as target molecules to discuss the effect of the incremental introduction of noble metals on SERRS activity. The distribution of hot spots was obtained by a finite-difference time-domain (FDTD) theoretical method, and the relevant SERRS enhancement mechanism was discussed. Given that Fe_3_O_4_ hollow spheres with large specific surface area have a good superparamagnetic property, CSSN NCs could be easily recovered through an external magnet, which provided potential possibilities of recycling and reuse in complex liquid environments [39]. Our work not only enriches research on the tailored design of ultra-sensitive SERRS substrates, but also realizes the rapid and quantitative detection of trace BPA, which may have many possible applications, such as in pollutant detection, environmental monitoring, and food safety.

## 2. Materials and Methods

### 2.1. Materials and Characterization

The details of this section can be found in Supplementary Materials.

### 2.2. Synthesis of PEI-DTC Aqueous Solution, Fe_3_O_4_ Hollow Spheres, Au Seeds, and Au@Ag Nanocrystals

PEI-DTC aqueous solution, Fe_3_O_4_ hollow spheres, Au seeds, and Au@Ag nanocrystals were obtained on the basis of our previous work [40].

### 2.3. Synthesis of Fe_3_O_4_-Au (FA) NCs

An amount of 20 mg of Fe_3_O_4_ hollow spheres were added to 20 mL of methanol, followed by dropwise addition of 25 mL of PEI-DTC aqueous solution. After the mixture stood for 60 min, the product was separated and gathered by a magnet. Then 20 mL of Au seeds were added under sonication. Finally, FA NCs were obtained after continuous sonication for 1 h and repeated washing.

### 2.4. Synthesis of FA@Ag@(Au@Ag) (CSSN) NCs

**Scheme 1:**
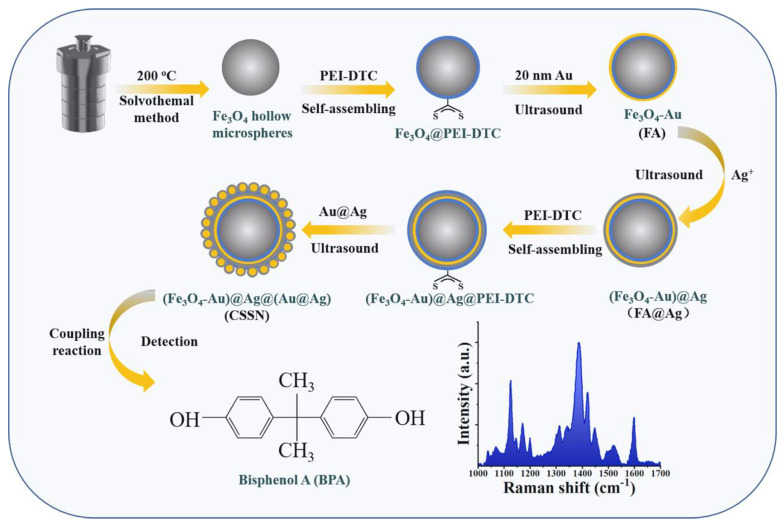
Scheme of synthetic process of CSSN NCs and SERRS detection protocol for BPA on CSSN SERRS substrates.

As illustrated in Scheme 1, the preparation process of CSSN NCs consists of two steps. The first step was the preparation of FA@Ag NCs. First, 10 mg of FA NCs and 5 mL of deionized water were mixed. Subsequently, AgNO_3_ (0.1 M; 3 mL) and reductant NH_2_OH·HCl (0.1 M; 12 mL) were added. FA@Ag NCs were obtained after sonication for 2 h and several wash cycles with ethanol.

The second step was the synthesis of CSSN NCs. The previously obtained FA@Ag NCs were blended with 20 mL of methanol, and 25 mL of PEI-DTC was dropwise-added. The mixture was stored for about 1 h. Then, FA@Ag@PEI-DTC NCs were obtained through washing and drying. After 10 mg of FA@Ag@PEI-DTC NCs was put into 5 mL of deionized water, 40 mL of Au@Ag nanocrystals was poured into the mixture and sonicated for about 2 h. Finally, the product was washed and dried to obtain CSSN NCs.

### 2.5. Pauly’s Reagents and Coupling Reaction

Three kinds of reagents were prepared and kept at 4 °C for further use. Reagent A was a mixture of *p*-aminobenzenesulfonic acid (4.5 g), HCl solution (12 M; 5 mL) and deionized water (500 mL). Reagent B was 5% NaNO_2_, and reagent C was 10% Na_2_CO_3_.

Coupling reaction: Reagents A + B + C + BPA ethanol solution in a volume ratio of 1:1:1:2.

### 2.6. FDTD Algorithm Method

Details of FDTD algorithm method can be found in Supplementary Materials.

### 2.7. SERRS Measurements

Before SERRS measurements, BPA was changed into azo dyes through a coupling reaction of BPA and diazonium ions to bind noble metals with high affinity and thus achieve highly sensitive detection of BPA. In this work, the phenol group of BPA could be converted into azo dyes with *p*-aminobenzenesulfonic acid through electrophilic aromatic substitution [41,42]. After the coupling reaction, BPA solution (25 µL) with different concentrations of 10^−10^ to 10^−4^ M and CSSN (1 mg) NCs were mixed in the aluminum pan, respectively. SERRS spectroscopy was performed under 514.5 nm laser and acquisition time was about 10 s.

## 3. Results and Discussion

### 3.1. Structure and Magnetic Properties of CSSN NCs

**Figure 1:**
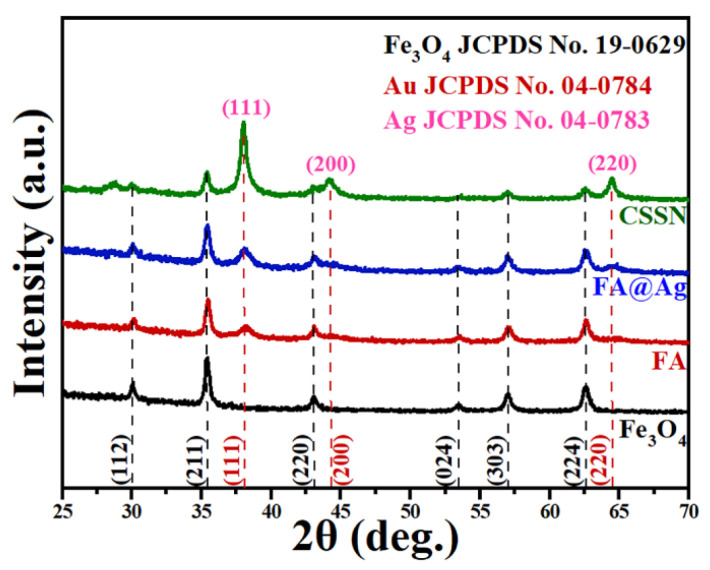
XRD pattern of Fe_3_O_4_ hollow spheres, FA, FA@Ag, and CSSN NCs.

XRD technology was used to study the structure and phase purity of Fe_3_O_4_ hollow spheres, FA, FA@Ag and CSSN NCs. As shown in Figure 1, the diffraction peaks of Fe_3_O_4_ hollow spheres located at 30.4°, 35.5°, 43.4°, 53.4°, 57.3°, and 62.8° refer to the (112), (211), (220), (024), (303), and (224) planes of Fe_3_O_4_, respectively, which can be indexed to the cubic inverse spinel structure of Fe_3_O_4_ (JCPDS 19-0629) [43,44]. In addition, the diffraction peaks are sharp and strong, which indicates that the prepared Fe_3_O_4_ hollow spheres have high phase purity and good crystallization. However, owing to the fact that Fe_3_O_4_ as well as γ-Fe_2_O_3_ have the identical spinel structure, it is not sufficient to identify them only by XRD results [45]. Therefore, the phase structure of Fe_3_O_4_ hollow spheres was further verified by Mössbauer spectroscopy. As depicted in Figure S1, the Mössbauer spectrum of Fe_3_O_4_ hollow spheres can be fitted into two sextets, and the magnetic sextets lines illustrate the typical double six peak structure of Fe_3_O_4_ [46,47]. The corresponding Mössbauer parameters of Fe_3_O_4_ hollow spheres are presented in Table S1. Hyperfine field is 48.7 and 45.5 Tesla, and the isomer displacement is 0.288 and 0.602 mm/s, which correspond to Fe^2+^ and Fe^3+^ at octahedral interstitial sites and Fe^3+^ at tetrahedral interstitial sites. After Au seeds were loaded on surfaces of Fe_3_O_4_ hollow spheres, four new XRD diffraction peaks emerged at 38.2°, 44.3°, and 64.5°, which were assigned to (111), (200), and (220) planes of Au (JCPDS 04-0784) [48]. It should be noted that the positions of Au and Ag characteristic peaks are too close to be distinguished [49]. Since the intensities of XRD diffraction peaks are related to the contents of phase in the mixture [50,51], the increase of the intensities of Ag/Au diffraction peaks in the XRD patterns of FA@Ag NCs proves that there is dense Ag adsorbed on the surfaces of FA NCs. The XRD pattern of CSSN NCs shows that the diffraction peaks intensity of Ag/Au further increases significantly when the Au@Ag nanocrystals are adhered to the FA@Ag NCs. By contrast, the XRD diffraction pattern of CSSN NCs exhibits weaker Fe_3_O_4_ characteristic peaks than that of Fe_3_O_4_ hollow spheres, FA, and FA@Ag NCs. This may be attributed to the declining proportion of Fe_3_O_4_ contents caused by the successful modification of the large amount of Au seeds and Au@Ag nanocrystals. Consequently, the information obtained from above XRD and Mössbauer analysis preliminary confirm the successful construction of Fe_3_O_4_ hollow spheres, FA, FA@Ag, and CSSN NCs.

For researching the magnetic properties of Fe_3_O_4_ hollow spheres, FA, FA@Ag, and CSSN NCs, their magnetic hysteresis (*M*-*H*) loops were tested via vibrating sample magnetometer (VSM), as displayed in Figure S2. The *M-H* loops show that Fe_3_O_4_ hollow spheres, FA, FA@Ag, and CSSN NCs have superparamagnetic property. The saturation magnetization (Ms) values of Fe_3_O_4_ hollow spheres, FA, FA@Ag, and CSSN NCs were 89.1, 74.5, 63.7, and 53.6 emu·g^−1^, respectively. It was found that the Ms value gradually reduces with the incremental introduction of noble metal due to the diamagnetism of noble metal nanocrystals [52]. As depicted in the inset of Figure S2, the CSSN NCs can be collected by an external magnet within 50 s even if their Ms value is the lowest among all the materials. The remarkable magnetic response property means that CSSN NCs have great convenience in rapid separation and detection in complex liquid environments.

**Figure 2:**
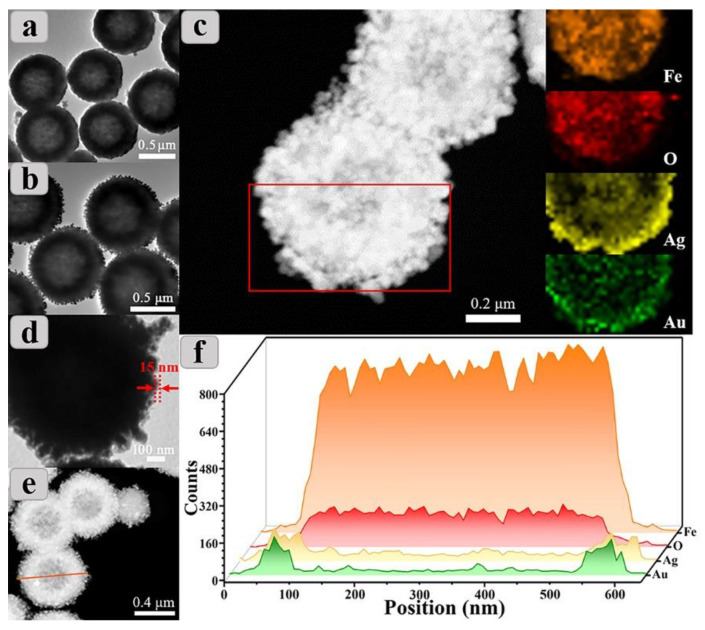
TEM images of Fe_3_O_4_ hollow spheres (**a**) and FA NCs (**b**). Dark-field TEM images and EDS elemental mapping results (O, Fe, Au and Ag) of FA@Ag NCs (**c**). TEM image of FA@Ag@PEI-DTC NCs (**d**). Dark-field TEM images (**e**) and EDS line scanning spectra of CSSN NCs (**f**).

The morphology, size, and structure of the obtained products were studied by TEM, EDS elemental mapping, and EDS line scanning. As depicted in Figure 2a, the Fe_3_O_4_ nanocrystals show uniform spherical shapes with size of about 600 nm, and the dark edges and shallow cores of Fe_3_O_4_ nanocrystals suggest the formation of hollow structures. It can be seen from the TEM image of FA NCs (Figure 2b) that Au seeds (about 20 nm) are densely and uniformly loaded on the surfaces of Fe_3_O_4_ hollow spheres. Subsequently, the seed-mediated growth technique was used to grow Ag shell on the surfaces of FA NCs. Specifically, Au seeds on Fe_3_O_4_ hollow spheres were employed as nucleation sites for the formation of Ag shells. As exhibited in the dark-field TEM image of FA@Ag NCs (Figure 2c), with the continuous reduction of Ag^+^ on surfaces of FA NCs, Ag shells with subtle roughness formed on the surfaces of FA NCs, and elements of Au and Ag are uniformly distributed on the surfaces of Fe_3_O_4_ hollow spheres, as shown in the corresponding EDS elemental mapping in Figure 2c. In order to create more hot spots, Au@Ag nanocrystals continued to be assembled on the surfaces of FA@Ag NCs to obtain CSSN NCs by PEI-DTC layers. As presented in Figure 2d, PEI-DTC layers with a thickness of around 15 nm are uniformly coated on the surfaces of FA@Ag NCs. For directly confirming the construction of core–shell–satellite structure in CSSN NCs, we took EDS line scanning profiles (Figure 2f) across CSSN NCs as presented by the orange highlighted line in the dark-field TEM image of Figure 2e. As shown in Figure 2f, it was found that the size of CSSN NCs increased to around 800 nm due to the presence of Au seeds, Ag shells, and Au@Ag nanocrystals, which is bigger than that of Fe_3_O_4_ hollow spheres. Moreover, the elements show a symmetrical distribution with the change of detection position. The X-ray intensity of Au and Ag is maximum while Fe and O is minimum in the edge region. By comparison, the relative intensity of Fe and O increases gradually and almost no X-ray intensity of Au and Ag is observed in the direction of the orange arrow. This proves that the Au@Ag nanocrystals are adsorbed firmly on the outermost layer of FA@Ag@PEI-DTC by the affinities between the bidentate ligands with two chelating sulfur groups and Au@Ag nanocrystals [31]. Therefore, the EDS line-scanning results of CSSN NCs are consistent with the conclusions of XRD, TEM, and corresponding EDS mapping, which proves that the formation of CSSN NCs with the core–shell–satellite structure is convincing.

**Figure 3:**
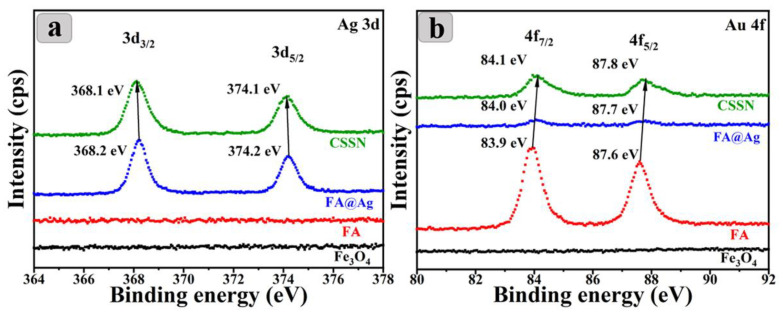
High-resolution XPS spectra of Fe_3_O_4_ hollow spheres, FA, FA@Ag, and CSSN NCs: Ag 3d (**a**) and Au 4f (**b**).

The valence of elements in CSSN NCs was determined by XPS technology. Full XPS spectra of Fe_3_O_4_ hollow spheres, FA, FA@Ag, and CSSN NCs are exhibited in Figure S3. Within detection limit of XPS, Fe 2p, O1s, Au 4f, Ag 3d, and C 1s were observed and no impurity was found. High-resolution XPS results of Ag 3d and Au 4f are reflected in Figure 3. Ag 3d spectra in Figure 3a display peaks at 368.2 and 374.2 eV with a spin-orbit splitting of 6 eV for CSSN NCs, which are attributable to characteristics of Ag 3d_3/2_ and Ag 3d_5/2_ of Ag^0^ [53]. As represented in Figure 3b, peaks of CSSN NCs at 84.1 and 87.8 eV with an energy difference of 3.7 eV are attributable to Au 4f_7/2_ and Au 4f_5/2_ of Au^0^ [54,55]. An interesting phenomenon is that the binding energy of Ag 3d as well as Au 4f changes slightly with the incremental introduction of noble metals. The positions of Ag 3d peaks of CSSN NCs are blue-shifted compared with FA@Ag NCs, and the positions of Au 4f peaks are red-shifted compared with FA@Ag and FA NCs. This shift in binding energy may be ascribed to the charge transfer from metallic Au to Ag [56,57,58].

### 3.2. Choice of Excitation Source

A widely accepted consensus is that the SERRS method can further improve the sensitivity of Raman scattering spectroscopy, which combines resonance enhancement and SERS [59]. Hence, it is very important to find a suitable excitation source to arouse the SERRS effect. UV–Vis spectra of BPA azo products, FA, FA@Ag, and CSSN NCs were tested to determine a light source with an appropriate wavelength that takes into account the resonance effect of BPA azo products and plasmon resonance effect of CSSN NCs with the laser. As presented in Figure S4, the absorption positions of BPA azo products, FA, FA@Ag, and CSSN NCs are located at 450, 545, 536, and 512 nm, respectively. Because the plasmonic resonance peak of CSSN NCs is closer to the absorption position of BPA azo products compared with FA and FA@Ag NCs, the coupling between BPA azo products and CSSN NCs is considerably easier. Therefore, 514.5 nm laser was selected as the excitation source in this work given that it is more suitable for the coupling absorption.

### 3.3. SERRS Spectra of BPA Azo Product on FA, FA@Ag, and CSSN NCs

**Figure 4:**
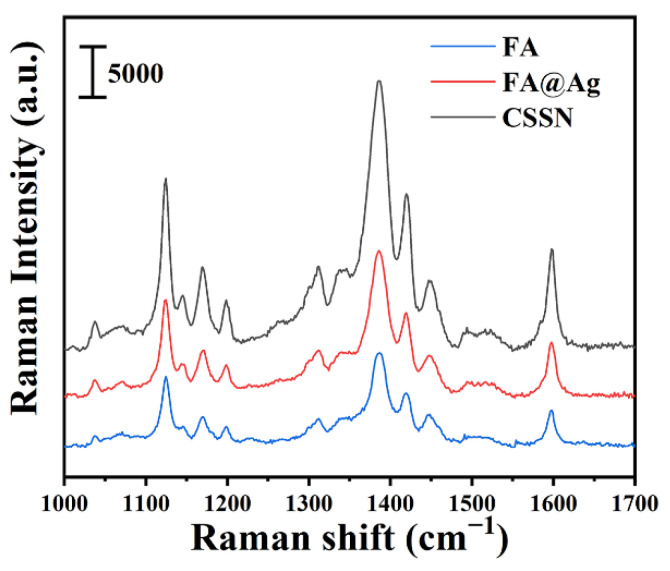
SERRS spectra after azo reaction of BPA azo products (10^−4^ M) on FA, FA@Ag, and CSSN NCs.

In order to directly evaluate the SERRS performance of various substrates, BPA (10^−4^ M) was chosen as the target molecule and FA, FA@Ag, and CSSN NCs served as SERRS substrates to explore their SERRS-enhancing capabilities, respectively. The detailed band assignments of BPA azo products are exhibited in Table S2 [37,60]. As reflected in Figure 4, with the incremental introduction of noble metals, the SERRS intensity of BPA gradually increases and CSSN NCs exhibit the strongest SERRS sensitivity compared with FA and FA@Ag NCs. The above phenomenon is foreseeable and will be discussed in detail below.

### 3.4. Mechanism of SERRS Enhancement

**Figure 5:**
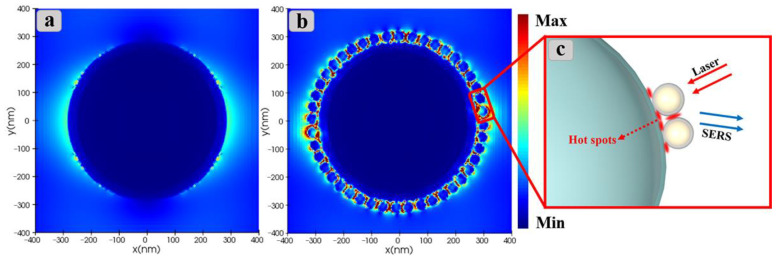
Electric field distribution of FA@Ag (**a**) and CSSN NCs (**b**) obtained by FDTD theoretical algorithm and the diagrammatic sketch of hot spots on CSSN NCs (**c**).

As mentioned above, the widely accepted theory for SERRS enhancement mechanism is EM mechanism, which derives from LSPR excitation of noble metal nanocrystals [61,62,63]. Compared with FA NCs, the surfaces of FA@Ag NCs are almost completely covered with Ag shells, which have better LSPR effect than Au, so it is reasonable that SERRS performance of FA@Ag NCs is somewhat better than that of FA NCs. In addition, strong electromagnetic fields will be excited in/between nearby noble metals because of the coupling effect and thus the SERRS signal intensity of target molecule can be significantly enhanced in hot spot regions [64,65,66]. Consequently, increasing the quantity of hot spots is an effective method to enhance the SERRS activity. To reveal why CSSN NCs have the highest SERRS enhancement, a FDTD theoretical algorithm was employed to visualize the distribution of electromagnetic field. As presented in Figure 5, it can be seen that more hot spots are generated on CSSN NCs. Compared with CSSN NCs, no obvious hot spots are found on the separate FA@Ag NCs (Figure 5a). A reasonable explanation is that the SERRS enhancement of FA@Ag NCs may come from hot spots generated by the aggregation of FA@Ag NCs in an actual detection procedure. As for CSSN NCs, large number of hot spots can also emerge in the region of narrow spacing between two adjacent Au@Ag nanocrystals, as shown in Figure 5b,c. In addition, there are large amounts of hot spots between Ag shells and outermost Au@Ag nanocrystals. It follows that the hot-spots effect is brought into full play through the construction of the core–shell–satellite structure. It also needs to be emphasized here that the introduction of the bimetallic Au@Ag nanocrystals is distinctly important, given that the Au@Ag nanocrystals make full use of excellent SERRS activity of Ag nanocrystals and high stability of Au nanocrystals [67,68]. Therefore, the excellent SERRS performance of CSSN NCs is attributed to a considerable quantity of hot spots generated by the core–shell–satellite structure, as well as excellent SERRS performance of Au@Ag nanocrystals.

### 3.5. Quantitative Detection of BPA Azo Products

**Figure 6:**
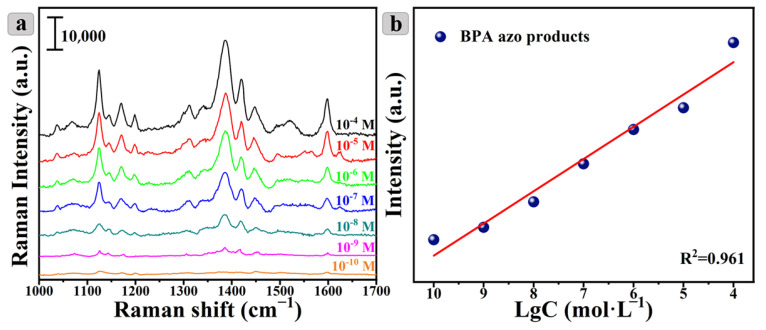
SERRS spectra of BPA azo products (from 10^−10^ to 10^−4^ M) adsorbed on CSSN NCs (**a**). The relationship between the SERRS intensity and concentrations of BPA azo products (**b**).

In order to assess the practicability of CSSN NCs as SERRS substrate for quantitative and sensitive detection of BPA, BPA azo products with different concentration from 10^−10^ to 10^−4^ M were chosen as probe molecules. Sharp and strong characteristic peaks of BPA azo products can be clearly observed from SERRS spectra illustrated in Figure 6a. SERRS intensities of BPA azo products rise monotonously with the increase of concentrations. The limit of detection (LOD) for the detection of BPA is as low as 10^−10^ M (about 0.023 ng/mL), which is well below the safety limit of the European Union (0.6 mg/kg), as well as China (10 ng/mL) [69]. More importantly, compared with the previous reports, our as-prepared CSSN substrate has the highest SERRS enhancement performance (Table 1) [70,71,72,73,74,75]. Moreover, the relationship between the concentrations of BPA azo products adsorbed on CSSN NCs and the corresponding SERRS intensities at 1384 cm^−1^ is reflected in Figure 6b. The linear relationship versus the logarithm of the concentrations and correlation coefficient (R^2^) is up to 0.96, which further proves that CSSN NCs are high performance SERRS sensors and can realize quantification of BPA down to 10^−10^ M.

## 4. Conclusions

In conclusion, magnetic core–shell–satellite CSSN NCs for ultra-sensitive SERRS detection of BPA have been successfully developed. The coupling reactions between BPA and Pauly’s reagents not only improved the affinity between BPA and substrates, but also amplified the SERRS signals due to the SERRS effect generated by the combination of resonance of BPA azo products and plasma resonance of noble metals. BPA azo products were chosen as target molecules to investigate the effect of incremental introduction of noble metals on SERRS activity. The distribution of electromagnetic field of CSSN NCs was studied through FDTD theoretical algorithm. The results revealed that a considerable number of hot spots were produced on the core–shell–satellite structure. The excellent SERRS activity of CSSN NCs was attributed to abundant hot spots of core–shell–satellite structure as well as outstanding SERRS activity of Au@Ag nanocrystals. BPA azo products were used to evaluate the practicability of CSSN NCs as SERRS substrate. When the concentrations of BPA azo products ranged from 10^−10^ to 10^−4^ M, SERRS intensities followed linear relationship versus the logarithm of the concentrations, and LOD was as low as 10^−10^ M. In addition, the Ms value of superparamagnetic CSSN NCs was 53.6 emu·g^−1^, which gave CSSN NCs the function of rapid separation and detection in complex liquid environments by an external magnetic field. This study not only provides a novel ultra-sensitive SERRS substrate, but also shows enormous potential for the field of food safety and environmental pollution control.

## Figures and Tables

**Table 1:** Comparison of the SERS sensitivity of different SERS substrates to BPA.

SERS Substrates	LOD (M)	References
MoS_2_/ZnO	10^−9^	[70]
Au/MBA/PEG/BADGE	3 × 10^−9^	[71]
Self-assembled graphitic substrates	10^−6^	[72]
Ag-thiolated β-cyclodextrin	10^−7^	[73]
MIPs@Ag NPs	5 × 10^−8^	[74]
Ag@MIP	10^−9^	[75]
CSSN	10^−10^	this work

## Data Availability

Not applicable.

## Supplementary Materials

The following supporting information can be downloaded at: https://www.mdpi.com/article/10.3390/nano12193322/s1, Figure S1: Mössbauer spectrum of Fe_3_O_4_ hollow spheres; Figure S2: Magnetic hysteresis (*M-H*) loops of Fe_3_O_4_ hollow spheres, FA, FA@Ag and CSSN NCs (The inset is photograph of CSSN NCs dispersed in deionized water before and after magnet separation); Figure S3: Full XPS spectra of Fe_3_O_4_ hollow spheres, FA, FA@Ag and CSSN NCs; Figure S4: UV-Vis spectra of BPA azo products, FA, FA@Ag and CSSN NCs; Table S1: Mössbauer spectrum parameters of Fe_3_O_4_ hollow spheres; Table S2: Band assignments in the SERRS spectra of BPA azo products.
