# Supplementary material for: Magnetic-Core–Shell–Satellite Fe3O4-Au@Ag@(Au@Ag) Nanocomposites for Determination of Trace Bisphenol A Based on Surface-Enhanced Resonance Raman Scattering (SERRS)
Source: Nanomaterials (Basel). 2022 Sep 24;12(19):3322. doi: 10.3390/nano12193322 (PMC9565892; doi:10.3390/nano12193322)
Supplement: Supplementary file 1 [file nanomaterials-12-03322-s001.zip › nanomaterials-1905140-supplementary.pdf]

Supplementary Materials

2.1 Materials and characterization

Iron chloride hexahydrate ($FeCl_3·6H_2O$) was purchased from Shanghai Macklin Biochemical Co., Ltd. (Shanghai, China). Potassium hydroxide (KOH), sodium citrate dihydrate ($Na_3C_6H_5O_7·2H_2O$), silver nitrate ($AgNO_3$), ethylene glycol (EG), sodium dodecyl sulfate (SDS), sodium acetate ($NaAc·3H_2O$), *p*-aminobenzenesulfonic acid, hydrochloric acid (HCl), sodium nitrite ($NaNO_2$), sodium carbonate (Na_2CO_3), hydroxylammonium chloride ($NH_2OH·HCl$) and absolute ethanol were purchased from Sinopharm Chemical Reagent Co, Ltd. (Shanghai, China). Bisphenol A (BPA), polyethyleneimine (PEI, branched, $M_W \approx 25000$ g/mol), gold chloride tetrahydrate ($HAuCl_4·4H_2O$), methanol (CH_3OH) and carbon disulfide (CS_2) were obtained from Aladdin Reagent Co., Ltd. (Shanghai, China). All chemicals used in this experiment were of analytical grade and without further purification.

The structure, morphology and elemental composition of the prepared samples were determined by X-ray diffractometer (XRD; Rigaku D/Max-2500) using Cu Kα radiation (λ =1.5406 Å), Mössbauer spectrum (Mössbauer; FAST Comtec Mössbauer system), scanning electron microscopy (SEM; JEOL JSM-7800F), X-ray photoelectron spectroscopy (XPS; Thermo Scientific ESCALAB 250Xi), energy dispersive spectrometer (EDS) and transmission electron microscopy analysis (TEM; JEOL 2100). Ultraviolet-visible spectra were measured with a spectrophotometer (UV-Vis; Shimadzu UV3600). The magnetic properties of the samples were obtained by vibrating sample magnetometer (VSM; Lakeshore cryotronics 730) at room temperature. The SERRS spectra were measured using a Renishaw Raman microsystem 2000 equipped with a charge-coupled device (CCD) detector and a holographic notch filter. An Ar^+ ion laser (Spectra Physics) with a wavelength of 514.5 nm was used as the excitation source for SERRS measurements.

2.6 FDTD algorithm method

The electromagnetic field enhancement of CSSN was simulated by using FDTD software (Lumerical solutions). The simulation region was an 800 × 800 × 800 nm^3 cuboid space surrounded by absorber layers to avoid numerical reflections. And the structural parameters in the FDTD simulation were similar to the observed values of the CSSN in the experiment. A continuous-wave source (λ= 514.5 nm) with size of 800 × 800 nm^2 was placed in the positive *z*-axis. The polarization direction of the source was along the *y*-axis.

Table S1. Mössbauer spectrum parameters of Fe_3O_4 hollow spheres.

	IS	Qs	HIN	HWHM	AREA
A	0.288	0.015	48.7	0.186	34.3
B	0.602	0.012	45.5	0.435	65.7

Table S2. Band assignments in the SERRS spectra of BPA azo products.

Wavenumber (cm^{-1})	Band assignments*
1198	ν(CN) + δ(CNN) (phenyl-N); δ(CH) + δ(OH) + ν(CC) from phenol groups
1311	δ(CCH) + δ(NCC) within phenyl rings
1384	ν(CC) within phenyl rings; δ(CH) + δ(OH) from phenol groups
1419	ν(NN)
1446	ν(NN) of trans isomers
1595	ν(CC) within phenol and/or phenyl rings

ν denotes stretching and δ denotes in-plane bending.

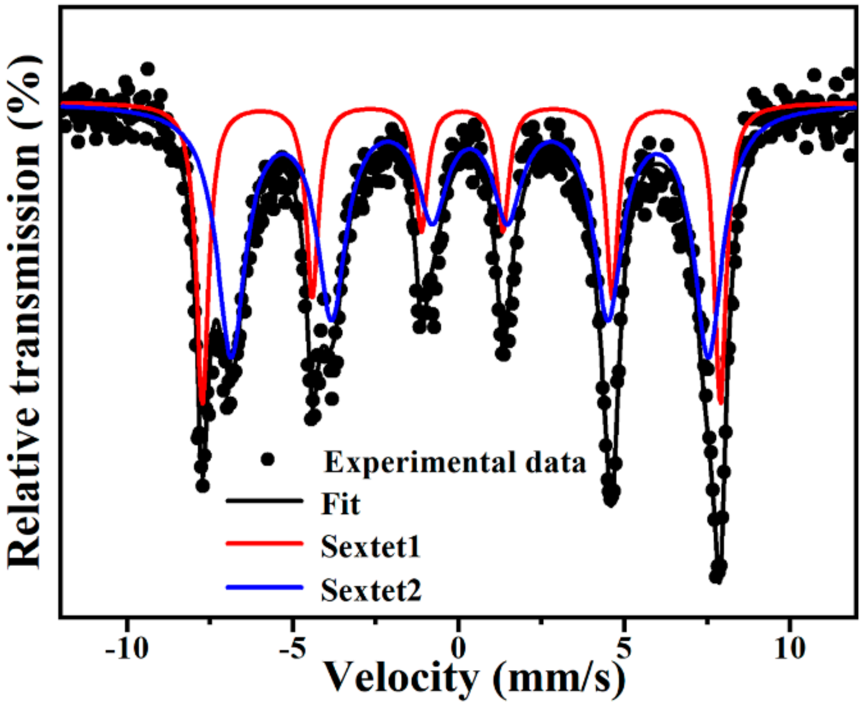

Figure S1. Mössbauer spectrum of Fe_3O_4 hollow spheres.

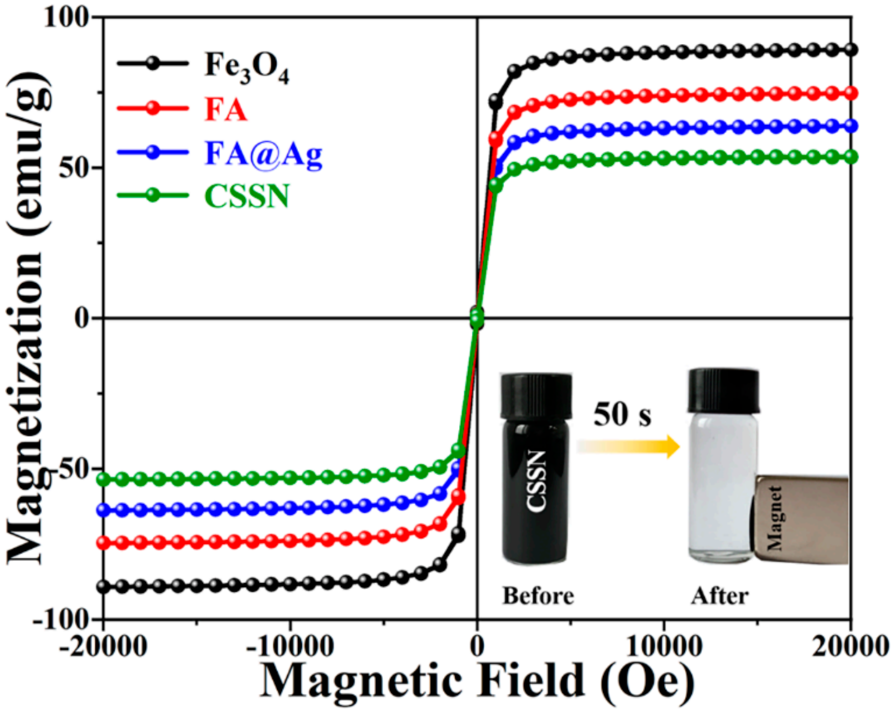

Figure S2. Magnetic hysteresis (*M-H*) loops of Fe_3O_4 hollow spheres, FA, FA@Ag and CSSN NCs (The inset is photograph of CSSN NCs dispersed in deionized water before and after magnet separation).

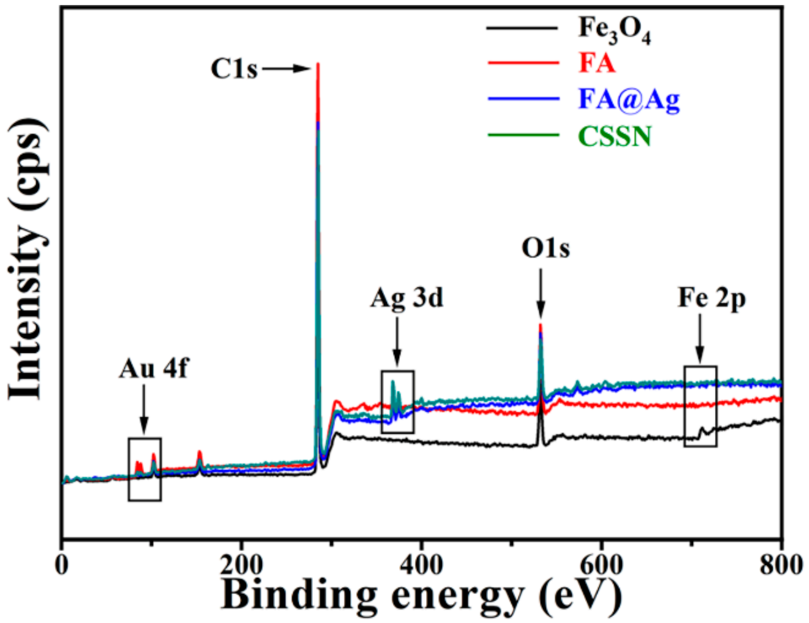

Figure S3. Full XPS spectra of Fe_3O_4 hollow spheres, FA, FA@Ag and CSSN NCs.

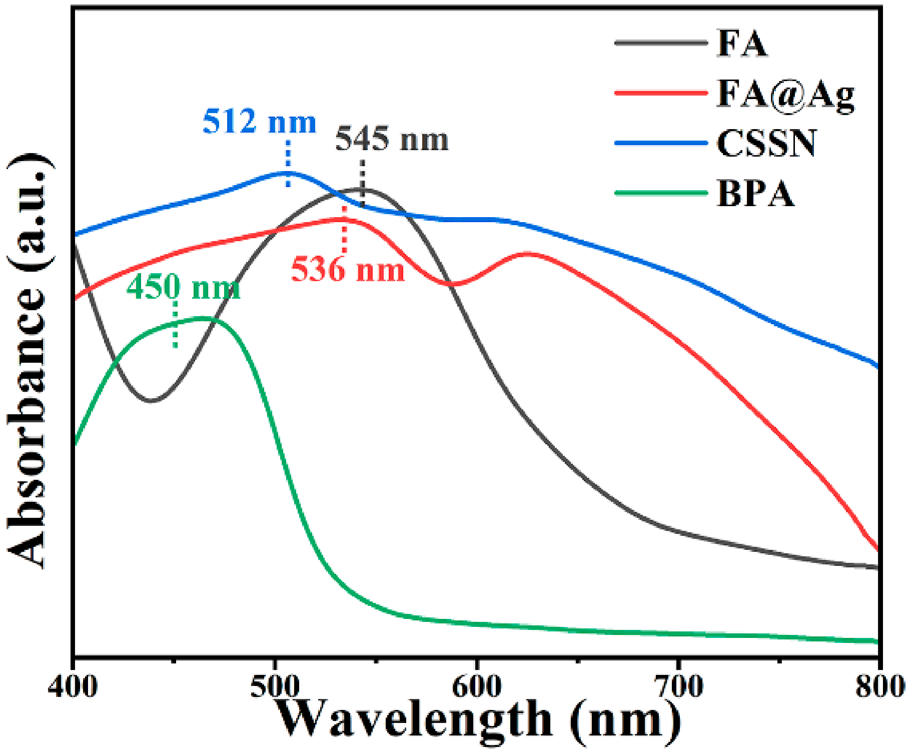

Figure S4. UV-Vis spectra of BPA azo products, FA, FA@Ag and CSSN NCs.
